# Tonsillotomy

**Published:** 1871-04

**Authors:** 


TONSILLOTOMY.—Billroth had lately to remove the left tonsil upon a hysterical lady. In doing so, he pulled the organ energetically towards the mesial line, so as to ensure a complete section. By this traction a fold of the pharyngeal mucous membrane was drawn out and cut with the tonsil. The bleeding was fearful, and the Professor considered that some large branch of the pharyngeal artery contained in the fold had been divided. The patient would not allow of the pressure of the finger within the mouth, so that compression was used on the carotid with success. Billroth warns surgeons concerning the too forcible drawing out of the tonsil.—*Lancet.*

